# Association between time of residence and self-perception of distress, interpersonal relationships, and social role in Venezuelan immigrants in Lima, Peru 2018–19: mixed-methods study

**DOI:** 10.1186/s12889-022-13459-4

**Published:** 2022-06-01

**Authors:** Frank Milton Delgado-Cáceres, Kevin Angel Silva-Parra, Paola A. Torres-Slimming

**Affiliations:** 1 School of Medicine, Faculty of Health Sciences, Universidad Peruana de Ciencias Aplicadas, Alameda San Marcos cuadra 2, Chorrillos, 15067 Lima, Peru; 2 Red Internacional América Latina, África, Europa, El Caríbe (ALEC) “Territorio(s), Poblaciones Vulnerables y Políticas Públicas.” Faculté des Lettres et Sciences Humaines, Université de Limoges, Limoges, France

**Keywords:** OQ-45.2, Venezuelan immigration, Immigrant distress, Immigration to Peru, Mixed methods

## Abstract

**Background:**

Immigrants arriving in a new country face changes that affect their social, employment, and migratory status. We carried out a mixed-methods study in the rapidly growing Venezuelan immigrant population in Lima, Peru. The objective was to determine whether there was an association between time in Peru and self-perception of symptom distress (SD), interpersonal relationships (IR), and social role (SR).

**Methods:**

The quantitative central component consisted of a cross-sectional study, surveying 152 participants using the Outcome Questionnaire 45.2 (OQ-45.2). The qualitative component, based on phenomenology, explored experiences and challenges during the migration process. Semi-structured in-depth interviews were conducted in 16 informants.

**Results:**

An association that was observed was the increase in the risk of clinically significant SR score with additional years of age. All informants mentioned having witnessed or experienced xenophobia in Peru. Every informant stated that significant labor differences existed between the countries. The most reported somatic symptoms were symptoms of anxiety and alterations of sleep. Additionally, no informant expressed a desire to remain in Peru long term.

**Conclusions:**

A minority of participants registered a clinically significant total score and in each of the three domains of SD, IR, and SR. No association between months in Lima and the self-perception of distress was found. However, this could be due to the short amount of time spent in Peru and any change in self-perception might only be perceived after years or decades spent in Peru. This study is one of the first to use mixed-methods to explore the mental health of the immigrant Venezuelan population.

## Introduction

Migration is defined by the World Health Organization (WHO) as ‘the movement of a person or a group of persons, either across an international border, or within a State’ [1]. Globally, the United Nations Department of Economic and Social Affairs estimated the number of international migrants in 2020 to be 281 million, an increase of 108 million since 2000, representing a 62% increase [2]. In light of the dramatic increase in the number of immigrants, the need for continuing studies in these populations, with emphasis in emerging populations, is essential.

One particular aspect that requires emphasis is mental health, especially in emerging populations such as Venezuelans in Peru. The mental health of an immigrant is affected due to the maladaptation process stemming from changes in relationships with their environment (support systems) and identity (customs, values, and idiosyncrasies) as they arrive in their destination country [3]. Accordingly, acculturation stress arises when the demands required for the adaptation to a new culture are not addressed by the immigrant. This situation may end up manifesting itself as anxiety and depressive disorders [4].

A systematic review found that the principal factors affecting the mental health of an immigrant are the alteration of social roles; loss of family and community support; and uncertainty regarding their migratory and employment status [5]. A cohort study carried out in Sweden found that refugees faced an increased risk for schizophrenia and other nonaffective psychiatric disorders when compared to non-refugee immigrants from similar regions and the native population [6]. However, as was reported in a study of immigrant communities in Canada, immigrants are also less likely to seek medical attention compared to the native population [7].

With respect to time since arrival in the destination country and the mental health of immigrants, it has been reported that immigrants with less time in the country are generally healthier and have lower mortality rates compared to the native population than those immigrants with more time in the country [5]. This so-called ‘immigrant paradox’ is based on the assumption that poorer populations, like the immigrant population, should have worse health status. However, it is just the opposite [8]. Although it has been reported that some migrant communities have high levels of psychotic disorders, it has also been shown that they have lower rates of mental health problems in general when compared to the native population [7]. The mental health of immigrants and the appearance of mental health pathologies still requires greater understanding regarding the development and onset of symptoms.

A narrative review of literature in English, Spanish, French, and Portuguese uncovered that other factors associated with mental health include low income, unemployment, female gender, higher age, lower socioeconomic status, not living with a partner, perceived discrimination, and negative life events [9].

A determinant frequently associated with mental health is interpersonal relationships. It has been consistently associated as a protective factor against depression, stress, and mental disorders [10–13]. On the other hand, having smaller social networks and fewer close relationships has been associated with the presence of depressive symptoms [14].

Another influential determinant on mental health is work role, a key component of SR, [15]. A meta-analysis found that psychosocial stressors in the work environment increased the risk of common mental disorders by 1.2 to 1.8 times, with high workload and an effort-reward imbalance being the most influential [16]. A systematic review showed that psychosocial stressors at work may increase the risk of depression, while social support in the work environment could significantly reduce the risk of depression [17]. In addition, the migration process causes high levels of emotional stress that affect the health of the individual and can manifest physically and psychologically [18].

Massive migration from Venezuela in the past decade has become important on a global public health scale [19]. According to statistics from the United Nations High Commissioner for Refugees, the number of Venezuelan immigrants and refugees has risen to 6.1 million since the beginning of the present migration wave in 2015–2016, depending on when each country began collecting data [20]. Among the most popular destination countries are Colombia, Peru, and Chile. At the moment of the recollection of quantitative data, Peru was the second most popular country for Venezuelan immigrants with 477,060, which accounted for 19.6% of the total Venezuelan immigrant population. This number has since risen to 1,286,000 as of March, 2022 [20].

Peru has not experienced a migration phenomenon of this magnitude since the Chinese and Japanese migration waves of the nineteenth century [21, 22]. As a result, migration policies aimed at immigrant health need to be reformulated to address the present phenomenon. Encouragingly, however, steps are being taken on an international level to ensure adequate reception of immigrants [23].

Due to the relative novelty of Venezuelan migration into Peru, its impact on mental health of Venezuelan immigrants, as well as its possible association with time has not been studied in depth. As such, a mixed-methods study was carried out to study the mental health of Venezuelan immigrants into Peru. The objectives of the quantitative portion were to determine the association between months of residence and the self-perception of mental health in Venezuelan immigrants into Peru, as well as describe factors influencing their mental health. Secondary objectives were to carry out an exploratory factor analysis and calculate the reliability of the Outcome Questionnaire 45.2 (OQ-45.2) The objectives of the qualitative portion were to describe experiences from the migration process and explore the perception of immigrants regarding the factors they relate to affect their mental health.

## Methodology

### Type of study

The study was an explanatory sequential mixed-methods study with a quantitative core component consisting of a cross-sectional analytic study which took place from December 2018 to April 2019 using convenience-based sampling. The complementary qualitative component consisted of semi-structured in-depth interviews using convenience and snowball sampling from the months of June to December 2019.

### Study area and community profile

The population of Venezuelan immigrants surveyed in this study were contacted in four areas of high Venezuelan population density. The most feasible high-density areas were determined to be commercial zones (A, B, C de-identified for confidentiality) and a migration permit regulatory office (D) in Lima. The three commercial zones have a high percentage of informal commerce and were selected based on convenience and accessibility.

### Quantitative component

In order to participate in this study, participants needed to meet criteria such as: entered Peru from January 1, 2014 onwards, be at least 18 years old, and lived in Peru for a period of at least one month.

### Sample size

Calculation of sample size was done using a comparison of proportions using data from a Chilean study utilizing the OQ-45.2, which measured mental health in Peruvian and Colombian immigrants. One of the averages used in the comparison was 48%, the proportion of Peruvian immigrants who scored in the clinically significant range [23]. The other average used came from an assumed scenario that 25% of Venezuelan immigrants would have a clinically significant score. From this comparison, a minimum sample size of 136 was calculated. After taking into account a 10% rejection rate, the number of participants necessary for this study was found to be 152. The confidence interval was set at 95% with power at 80%.

#### Sampling

Potential participants were approached in a convenience-based sampling manner. The participants were given the option to answer the OQ-45.2 and sociodemographic items on their own or to have them read verbally by the investigators. Participants were free to leave at any point during this sequence and request the deletion of any part or the entirety of their participation.

### Variable definitions

The outcome variable was the categorization of OQ-45.2 global score into clinically significant or not clinically significant symptoms using a cut-off point of 73, as set by the Spanish language validation [24]. The OQ-45.2 is comprised of three domains: Symptomatic Distress (SD), Social Role (SR), and Interpersonal Relations (IR) [25]. Similarly, their respective cut-off points, as set by the Spanish language validation, of 43, 14, and 16 were used to categorize participants [24].

The main independent variable was time since arrival in Peru, measured in months. Other independent variables were relevant sociodemographic traits such as age group, gender, education level, civil status, and monthly income. The definition utilized by the WHO for late adolescent, young adult, and adult was used to categorize participants into age groups [26]. Monthly income was dichotomized based on the minimum wage of 930 Peruvian soles [27]. Migratory status was dichotomized on the basis of ability to legally acquire a job. Civil status was dichotomized on the basis of having a partner or not. The home states, defined as one of the 23 states of Venezuela plus the Capital District, of participants were collected and then categorized into nine administrative regions according to the designation set forth by the Venezuelan government on June 7, 1969 [28].

### Quantitative data analysis

A database of the surveys was digitized by two researchers in an independent manner. These two databases were compared; in the case of a discrepancy, the original survey was referenced and relevant corrections were made to the database. This process was repeated until there were no discrepancies. The resulting database was used for all analysis. Descriptive analysis was reported using frequencies and percentages for categorical variables, while numeric variables were reported using median and interquartile range. Bivariate analysis of two categorical variables (e.g, gender and dichotomization of the OQ-45.2) depended on expected values. If more than 20% of expected values were below five, Fisher’s exact test was used; otherwise, chi-squared test was used.

Bivariate analysis of a categorical variable (i.e., dichotomization of the OQ-45.2) with a numeric variable (time of residence in Peru, as measured in months) was based on assumptions of normal distribution and homogeneity of variance. Normal distribution, tested via the Shapiro-Wilk test, did not hold true for any of the analyses. Consequently, Wilcoxon rank-sum test was used for all bivariate analyses including a numeric variable. A *p*-value below 0.050 was considered significant. Multivariate analysis was done using robust Poisson regression.

Exploratory factor analysis.

Reliability analysis was also carried out to evaluate the internal consistency of the OQ-45.2 in the Venezuelan population of Lima.

### Qualitative component

#### Conceptual framework

Phenomenology was used, originating from the desire to explore and understand the experiences of immigrants during the process of migration and how they related to mental health [29]. A description about the phenomenon was done through in-depth interviews. The guiding research question was: What do Venezuelan immigrants in Peru experience during the migration process, and how do they self-perceive difficulties stemming from social roles, interpersonal relationships, and symptomatic distress?

#### Data collection

The qualitative supplemental section was done during the months of June to December 2019. The technique used was in-depth interview. The in-depth interview continued until the saturation point was reached [30]. The investigative team consisted of two investigators and an advisor qualified in mixed-method studies. The investigators were final-year medical students and received four three-hour training sessions over a period of 2 weeks conducted by the qualified advisor.

An in-depth interview is a strategy used in qualitative research that permits one-on-one interaction between the interviewer and informant. Discussion is led by the informant; the role of the interviewer is to initiate discussion in the topic. The direction of any further discussion depends on the informant. The information gathered are not facts, but rather an interpretation of stories [31].

The in-depth interviews were semi-structured in nature. A guide that touched upon social role, interpersonal relations, and symptom distress was designed to initiate conversation. The guide was first tested for comprehension on two informants which were not included in the sample size. Informants were free to delve into topics they found relevant, while the investigators were also free to explore issues that would enrich the conversation with further investigation. A total of 16 Venezuelan immigrants were interviewed and those interviews were recorded, either by hand or with an audio recorder. A total of 540 minutes were recorded from these interviews.

#### Informant selection

Informants were selected using convenience sampling and snowball sampling. Venezuelan contacts were initially approached to confirm their interest in participating in an in-depth interview. Informed consent was obtained in the same manner as the quantitative component. Finally, informants were asked for explicit permission to record answers on an audio recorder. Informants were free to leave at any point during this sequence as well as to request the deletion of any or all of their audio recording.

At the end of the interview, the informant was asked if they knew anyone else who was also a Venezuelan immigrant and who would be interested in participating in the study. If so, the individual was contacted; if not, possible informants were contacted using convenience-based sampling.

#### Qualitative analysis

Once the in-depth interviews were carried out, textual transcriptions were created on Microsoft Word and the audio recordings were deleted. From this point, themes were revised, and categories and subcategories were selected. To provide rigorous data analysis, the Colaizzi method was followed. This process included the following steps: i) all investigators separately familiarized themselves with the textual transcriptions, ii) significant codes were highlighted, iii) meanings were formulated and significant themes were outlined, iv) the transcript was read and the audio records were repeatedly listened to in order for further themes to be developed, v) central themes were decided upon and ranked around immigration experiences and mental health, vi) exhaustive condensation of descriptions and analysis from the experiences of the participants were structured to respond to the research objectives [32].

#### Mixed-methods analysis

According to the explorative nature of the mixed-method research, results from both epistemological data were selected in order to compare ideas and divergent and convergent ideas [32].

#### Ethics

This research project was approved by the Ethics Committee Board of the Peruvian University of Applied Sciences (ID: PI 111–18). All methods were performed in accordance with the Declaration of Helsinki. All participants were free to leave at any moment as well as request deletion of any part or the entirety of their participation. In both the qualitative and quantitative components, informed consent was verbally explained and the participant given a physical copy to read and accept.

## Results

### Quantitative results

#### Participant demographics

A total of 152 Venezuelan immigrants were surveyed in Lima. Participants were contacted in the selected areas of A (17.1%), B (15.8%), C (5.9%), and D (61.2%). Of these 152 participants, two were excluded when they abandoned the study. A further seven participants were excluded due to the percentage of blanks in any of the three domains exceeding 10%. At the conclusion of these steps, a total of 143 participants entered analysis.

Nearly 60% of immigrant participants were women. In terms of auto-reported race, more than a third of the population was ‘white’, while the ‘colored’ demographic made up nearly three-fifths of the population. Adults between the ages of 25 and 59 accounted for approximately 60% of participants. From the total survey, nearly 75% of participants had no partner. Regarding education level, almost two-thirds of the surveyed participants responded as having reached higher education. Only one out every 20 individuals did not have a Temporary Permanence Permit (TPP) and, thus, could not legally work. Individuals earning at least minimum wage were 75% as seen in Table 1.

**Table 1:** Sociodemographic characteristics of the surveyed Venezuelan immigrants in commercial zones of Lima, Peru (*N* = 143)

Variables	n	(%)
**Gender**		
Masculine	60	(42.0%)
Femenine	83	(58.0%)
**Age (median, IQR)**	26	(22–33)
**Age group**^a^		
18–19	8	(5.6%)
20–24	48	(33.6%)
25–59	87	(60.8%)
**Civil status**^b^		
Without partner	107	(74.8%)
With partner	36	(25.2%)
**Education level**		
Primary	3	(2.1%)
Secondary	48	(32.6%)
Higher education^c^	92	(64.3%)
**Race**^d^		
White	51	(35.7%)
Colored	81	(56.6%)
Black	7	(4.9%)
Indigenous	1	(0.7%)
Other	3	(2.1%)
**Monthly income (soles)**^e^		
< 930	40	(28.0%)
≥ 930	103	(72.0%)
**Months in Peru (median, IQR)**	7	(4 – 10)
**Migratory quality**^f^		
Without work permit	8	(5.6%)
With work permit	135	(94.4%)
**First entry point into Peru**		
Lima/Callao	14	(9.9%)
Piura	12	(8.5%)
Tumbes	115	(81.6%)
**Home region**		
Andean	12	(8.6%)
Capital	36	(25.9%)
Central	31	(22.3%)
Guayana	7	(5.0%)
Insular	4	(2.9%)
Llanos	3	(2.2%)
Eastern	16	(11.5%)
Central-Western	20	(14.4%)
Zulian	10	(7.2%)
**Travel method**		
Foot	1	(0.7%)
Bus	123	(86.0%)
Air	14	(9.8%)
Mixed^h^	5	(3.5%)

^a^Age groups are according to the World Health Organization, 2017

^b^With partner includes married (13.3%) and cohabitant (11.9%); without partner includes single (74.1%), and widowed (0.7%)

^c^Higher education includes university, institute, technical career, without importance to whether it was finalized

^d^Based on designations in the 2011 Venezuelan Census. Other refers to any other self-reported race that is not explicitly mentioned

^e^The minimum wage in Peru is 930 soles (April 1, 2018)

^f^Without work permit incudes refugee claimant (2.1%) and tourist (3.5%); with work permit includes Temporary Permit of Permanence (85.3%), resident (2.1%), family of resident (0.7%), refugee (2.8%), and immigrant (3.5%)

^g^Political-administrative regions are according to the Government of Venezuela (June 7, 1969)

^h^Any combination of more than one travel method

### Migratory status

The median time since arrival in Peru was 7 months, with an interquartile range from 4 to 10 months. The most common entry point into Peru was the Tumbes region, accounting for 80% of entries into the country. Immigrants reported predominantly coming from the Capital (25.9%) and Central (22.3%) regions of Venezuela, respectively. Other regions with significant populations were the Central-Western and Eastern regions.

With respect to travel method, the most common was bus, accounting for much of the population (90%). Other travel methods included via air (9.8%), mixed route (3.5%), and by foot (0.7%) as presented in Table 1.

### Description of the outcome variable (instrument)

The global score for the OQ-45.2 was clinically significant in only six participants (4.2%). In the SD domain, four participants (2.8%) registered clinically significant scores. As can be seen in Table 2, in the domains of IR and SR, 23.1 and 13.3% participants scored in the clinically significant range, respectively.

**Table 2:** Description of the OQ-45.2 results obtained in the surveyed Venezuelan immigrants in commercial zones of Lima, Peru (*N* = 143)

Variables	n	(%)
**Dichotomized total score (0–180)**		
No clinically significant symptoms	137	(95.8%)
Clinically significant symptoms (≥73)	6	(4.2%)
**Dichotomized Symptom Distress score (0–100)**		
No clinically significant symptoms	139	(97.2%)
Clinically significant symptoms (≥43)	4	(2.8%)
**Dichotomized Interpersonal Relationships score (0–44)**		
No clinically significant symptoms	124	(86.7%)
Clinically significant symptoms (≥16)	19	(13.3%)
**Dichotomized Social Role Score (0–36)**		
No clinically significant symptoms	110	(76.9%)
Clinically significant symptoms (≥14)	33	(23.1%)

### Outcome bivariate analysis

In the bivariate analysis, gender was associated with clinically significant overall OQ-45.2 score. In addition, age was associated with clinically significant SD score, while civil status was associated with clinically significant IR score. The associations between the total and domain scores with socio-demographic variables can be seen in Tables 3, 4, 5 and 6.

**Table 3:** Bivariate analysis of the dichotomized total OQ-45.2 score in the surveyed Venezuelan immigrants in commercial zones of Lima, Peru (*N* = 143)

Variables	Clinically Significant		Not Clinically Significant		***p***-value*
	(*n* = 6)		(*n* = 137)		
**Gender**					
Masculine	6	(7.2%)	77	(92.8%)	***p*** **= 0.040**
Feminine	0	(0.0%)	60	(100.0%)	
**Age (median, IQR)**	24	(21 – 27)	26	(22 – 33)	*p* = 0.270†
**Age group**^a^					
18–19	1	(12.5%)	7	(87.5%)	*p* = 0.427
20–24	2	(4.2%)	46	(95.8%)	
25–59	3	(3.5%)	84	(96.5%)	
**Civil status**^b^					
Without partner	6	(5.6%)	101	(94.4%)	*p* = 0.337
With partner	0	(0.0%)	36	(100.0%)	
**Education level**					
Primary	0	(0.0%)	3	(100.0%)	*p* = 0.280
Secondary	4	(8.3%)	44	(91.7%)	
Higher education^c^	2	(2.2%)	90	(97.8%)	
**Race**^d^					
White	0	(0.0%)	51	(100.0%)	*p* = 0.235
Colored	6	(7.4%)	75	(92.6%)	
Black	0	(0.0%)	7	(100.0%)	
Indigenous	0	(0.0%)	1	(100.0%)	
Other	0	(0.0%)	3	(100.0%)	
**Monthly income (soles)**^e^					
< 930	2	(5.0%)	38	(95.0%)	*p* = 0.672
≥ 930	4	(3.9%)	99	(96.1%)	
**Months in Peru (median, IQR)**	6	(3 – 9)	7	(4 – 10)	*p* = 0.701†
**Migratory quality**^f^					
Without work permit	0	(0.0%)	8	(100.0%)	*p* = 1.000
With work permit	6	(4.4%)	129	(95.6%)	
**First entry point into Peru**					
Lima/Callao	0	(0.0%)	14	(100.0%)	*p* = 1.000
Piura	0	(0.0%)	12	(100.0%)	
Tumbes	6	(5.2%)	109	(94.8%)	
**Home region**					
Andean	0	(0.0%)	12	(100.0%)	*p* = 0.542
Capital	2	(5.6%)	34	(94.4%)	
Central	1	(3.2%)	30	(96.8%)	
Guayana	1	(14.3%)	6	(85.7%)	
Insular	0	(0.0%)	4	(100.0%)	
Llanos	0	(0.0%)	3	(100.0%)	
Eastern	2	(12.5%)	14	(87.5%)	
Central-Western	0	(0.0%)	20	(100.0%)	
Zulia	0	(0.0%)	10	(100.0%)	
**Travel method**					
Foot	0	(0.0%)	1	(100.0%)	*p* = 0.319
Bus	5	(4.1%)	118	(95.9%)	
Air	0	(33.3%)	14	(100.0%)	
Mixed^h^	1	(20.0%)	4	(80.0%)	

*p < 0.050 using Fisher exact test except those marked with † or †† where Mann-Whitney U test or chi-squared test were used, respectively

^a^Age groups are according to the World Health Organization, 2017

^b^With partner includes married (13.3%) and cohabitant (11.9%); without partner includes single (74.1%), and widowed (0.7%)

^c^Higher education includes university, institute, technical career, without importance to whether it was finalized

^d^Based on designations in the 2011 Venezuelan Census. Other refers to any other self-reported race that is not explicitly mentioned

^e^The minimum wage in Peru is 930 soles (April 1, 2018)

^f^Without work permit incudes refugee claimant (2.1%) and tourist (3.5%); with work permit includes Temporary Permit of Permanence (85.3%), resident (2.1%), family of resident (0.7%), refugee (2.8%), and immigrant (3.5%)

^g^Political-administrative regions are according to the Government of Venezuela (June 7, 1969)

^h^Any combination of more than one travel method

**Table 4:** Bivariate analysis of the dichotomized Symptom Distress score in the surveyed Venezuelan immigrants in commercial zones of Lima, Peru (*N* = 143)

Variables	Clinically Significant		Not Clinically Significant		***p***-value*
	(*n* = 4)		(*n* = 139)		
**Gender**					
Masculine	2	(3.3%)	81	(97.6%)	*p* = 1.000
Feminine	2	(2.4%)	58	(96.7%)	
**Age (median, IQR)**	22.5	(18 – 33)	26	(22 – 33)	*p* = 0.303†
**Age group**^a^					
18–19	2	(25.0%)	6	(75.0%)	***p*** **= 0.009**
20–24	0	(0.0%)	48	(100.0%)	
25–59	2	(2.3%)	85	(97.7%)	
**Civil status**^b^					
Without partner	3	(2.8%)	104	(97.2%)	*p* = 1.000
With partner	1	(2.8%)	35	(97.2%)	
**Education level**					
Primary	0	(0.0%)	3	(100.0%)	*p* = 0.639
Secondary	2	(4.2%)	46	(95.8%)	
Higher education^c^	2	(2.2%)	90	(97.8%)	
**Race**^d^					
White	1	(2.0%)	50	(98.0%)	*p* = 1.000
Colored	3	(3.7%)	78	(96.3%)	
Black	0	(0.0%)	7	(100.0%)	
Indigenous	0	(0.0%)	1	(100.0%)	
Other	0	(0.0%)	3	(100.0%)	
**Monthly income (soles)**^e^					
< 930	1	(2.5%)	39	(97.5%)	*p* = 1.000
≥ 930	3	(2.9%)	100	(97.1%)	
**Months in Peru (median, IQR)**	4.5	(2–8.5)	7	(4–10)	*p* = 0.320†
**Migratory quality**^f^					
Without work permit	0	(0.0%)	8	(100.0%)	*p* = 1.000
With work permit	4	(3.0%)	131	(97.0%)	
**First entry point into Peru**					
Lima/Callao	1	(7.1%)	13	(92.9%)	*p* = 0.155
Piura	1	(8.3%)	11	(91.7%)	
Tumbes	2	(1.7%)	113	(98.3%)	
**Home region**					
Andean	0	(0.0%)	12	(100.0%)	*p* = 0.071
Capital	0	(0.0%)	36	(100.0%)	
Central	1	(3.2%)	30	(96.8%)	
Guayana	2	(28.6%)	5	(71.4%)	
Insular	0	(0.0%)	4	(100.0%)	
Llanos	0	(0.0%)	3	(100.0%)	
Eastern	0	(0.0%)	16	(100.0%)	
Central-Western	1	(5.0%)	19	(95.0%)	
Zulia	0	(0.0%)	10	(100.0%)	
**Travel method**					
Foot	0	(0.0%)	1	(100.0%)	*p* = 0.071
Bus	2	(1.6%)	121	(98.4%)	
Air	1	(7.1%)	13	(92.9%)	
Mixed^h^	1	(20.0%)	4	(80.0%)	

*p < 0.050 using Fisher exact test except those marked with † or †† where Mann-Whitney U test or chi-squared test were used, respectively

^a^Age groups are according to the World Health Organization, 2017

^b^With partner includes married (13.3%) and cohabitant (11.9%); without partner includes single (74.1%), and widowed (0.7%)

^c^Higher education includes university, institute, technical career, without importance to whether it was finalized

^d^Based on designations in the 2011 Venezuelan Census. Other refers to any other self-reported race that is not explicitly mentioned

^e^The minimum wage in Peru is 930 soles (April 1, 2018)

^f^Without work permit incudes refugee claimant (2.1%) and tourist (3.5%); with work permit includes Temporary Permit of Permanence (85.3%), resident (2.1%), family of resident (0.7%), refugee (2.8%), and immigrant (3.5%)

^g^Political-administrative regions are according to the Government of Venezuela (June 7, 1969)

^h^Any combination of more than one travel method

**Table 5:** Bivariate analysis of the dichotomized Interpersonal Relationships score in the surveyed Venezuelan immigrants in commercial zones of Lima, Peru (*N* = 143)

Variables	Clinically Significant		Not Clinically Significant		***p***-value*
	(*n* = 19)		(*n* = 124)		
**Gender**					
Masculine	13	(15.7%)	70	(84.3%)	*p* = 0.455
Feminine	6	(10.0%)	54	(90.0%)	
**Age (median, IQR)**	22	(20 – 28)	26.5	(23 – 35)	***p*** **= 0.011**†
**Age group**^a^					
18–19	2	(25.0%)	6	(75.0%)	*p* = 0.177††
20–24	9	(18.8%)	39	(81.2%)	
25–59	8	(9.2%)	79	(90.8%)	
**Civil status**^b^					
Without partner	18	(16.8%)	89	(83.2%)	***p*** **= 0.044**
With partner	1	(2.8%)	35	(97.2%)	
**Education level**					
Primary	0	(0.0%)	3	(100.0%)	*p* = 0.420
Secondary	9	(18.8%)	39	(81.2%)	
Higher education^c^	10	(10.9%)	82	(89.1%)	
**Race**^d^					
White	3	(5.9%)	48	(94.1%)	*p* = 0.249
Colored	15	(18.5%)	68	(81.5%)	
Black	1	(14.3%)	6	(85.7%)	
Indigenous	0	(0.0%)	1	(100.0%)	
Other	0	(0.0%)	3	(100.0%)	
**Monthly income (soles)**^e^					
< 930	5	(12.5%)	35	(87.5%)	*p* = 0.863††
≥ 930	14	(13.6%)	89	(86.4%)	
**Months in Peru (median, IQR)**	6	(3 – 9)	7	(4–10)	*p* = 0.623†
**Migratory quality**^f^					
Without work permit	2	(25.0%)	6	(75.0%)	*p* = 0.288
With work permit	17	(12.6%)	118	(87.4%)	
**First entry point into Peru**					
Lima/Callao	2	(14.3%)	12	(85.7%)	*p* = 0.505
Piura	0	(0.0%)	12	(100.0%)	
Tumbes	17	(14.8%)	98	(85.2%)	
**Home region**					
Andean	2	(16.7%)	10	(83.3%)	*p* = 0.801
Capital	3	(8.3%)	33	(91.7%)	
Central	5	(16.1%)	26	(83.9%)	
Guayana	2	(28.6%)	5	(71.4%)	
Insular	0	(0.0%)	4	(100.0%)	
Llanos	0	(0.0%)	3	(100.0%)	
Eastern	3	(18.8%)	13	(81.2%)	
Central-Western	2	(10.0%)	18	(90.0%)	
Zulia	2	(20.0%)	8	(90.0%)	
**Travel method**					
Foot	0	(0.0%)	1	(100.0%)	*p* = 0.754
Bus	16	(13.0%)	107	(87.0%)	
Air	2	(14.3%)	12	(85.7%)	
Mixed^h^	1	(20.0%)	4	(80.0%)	

*p < 0.050 using Fisher exact test except those marked with † or †† where Mann-Whitney U test or chi-squared test were used, respectively

^a^Age groups are according to the World Health Organization, 2017

^b^With partner includes married (13.3%) and cohabitant (11.9%); without partner includes single (74.1%), and widowed (0.7%)

^c^Higher education includes university, institute, technical career, without importance to whether it was finalized

^d^Based on designations in the 2011 Venezuelan Census. Other refers to any other self-reported race that is not explicitly mentioned

^e^The minimum wage in Peru is 930 soles (April 1, 2018)

^f^Without work permit incudes refugee claimant (2.1%) and tourist (3.5%); with work permit includes Temporary Permit of Permanence (85.3%), resident (2.1%), family of resident (0.7%), refugee (2.8%), and immigrant (3.5%)

^g^Political-administrative regions are according to the Government of Venezuela (June 7, 1969)

^h^Any combination of more than one travel method

**Table 6:** Bivariate analysis of the dichotomized Social Role score in the surveyed Venezuelan immigrants in commercial zones of Lima, Peru (*N* = 143)

Variables	Clinically Significant		Not Clinically Significant		***p***-value*
	(*n* = 33)		(*n* = 110)		
**Gender**					
Masculine	22	(26.5%)	61	(73.5%)	*p* = 0.316
Feminine	11	(18.3%)	49	(81.7%)	
**Age (median, IQR)**	28	(24 – 39)	26	(22–32)	*p* = 0.072†
**Age group**^a^					
18–19	1	(12.5%)	7	(87.5%)	*p* = 0.458††
20–24	9	(18.8%)	39	(81.2%)	
25–59	23	(26.4%)	64	(73.6%)	
**Civil status**^b^					
Without partner	24	(22.4%)	83	(77.6%)	*p* = 0.752††
With partner	9	(25.0%)	27	(75.0%)	
**Education level**					
Primary	1	(33.3%)	2	(66.7%)	*p* = 0.141
Secondary	15	(31.3%)	33	(68.7%)	
Higher education^c^	17	(18.5%)	75	(81.5%)	
**Race**^d^					
White	9	(17.7%)	42	(82.3%)	*p* = 0.687
Colored	21	(25.9%)	60	(74.1%)	
Black	2	(28.6%)	5	(71.4%)	
Indigenous	0	(0.0%)	1	(100.0%)	
Other	1	(33.3%)	2	(66.7%)	
**Monthly income (soles)**^e^					
< 930	31	(77.5%)	9	(22.5%)	*p* = 0.919††
≥ 930	24	(23.3%)	79	(76.7%)	
**Months in Peru (median, IQR)**	7	(3 – 11)	6	(4 – 9)	*p* = 0.686†
**Migratory quality**^f^					
Without work permit	0	(0.0%)	8	(100.0%)	*p* = 0.198
With work permit	33	(24.4%)	102	(75.6%)	
**First entry point into Peru**					
Lima/Callao	1	(7.1%)	13	(92.9%)	*p* = 0.221
Piura	4	(33.3%)	8	(66.7%)	
Tumbes	28	(24.1%)	88	(75.9%)	
**Home region**					
Andean	1	(8.3%)	11	(91.7%)	*p* = 0.477
Capital	10	(27.8%)	26	(72.2%)	
Central	10	(32.3%)	21	(67.7%)	
Guayana	2	(28.6%)	5	(71.4%)	
Insular	1	(25.0%)	3	(75.0%)	
Llanos	1	(33.3%)	2	(66.7%)	
Eastern	3	(18.8%)	13	(81.2%)	
Central-Western	5	(25.0%)	15	(75.0%)	
Zulia	0	(0.0%)	10	(100.0%)	
**Travel method**					
Foot	0	(0.0%)	1	(100.0%)	*p* = 0.311
Bus	32	(26.0%)	91	(74.0%)	
Air	1	(7.1%)	13	(92.9%)	
Mixed^h^	0	(0.0%)	5	(100.0%)	

*p < 0.050 using Fisher exact test except those marked with † or †† where Mann-Whitney U test or chi-squared test were used, respectively

^a^Age groups are according to the World Health Organization, 2017

^b^With partner includes married (13.3%) and cohabitant (11.9%); without partner includes single (74.1%), and widowed (0.7%)

^c^Higher education includes university, institute, technical career, without importance to whether it was finalized

^d^Based on designations in the 2011 Venezuelan Census. Other refers to any other self-reported race that is not explicitly mentioned

^e^The minimum wage in Peru is 930 soles (April 1, 2018)

^f^Without work permit incudes refugee claimant (2.1%) and tourist (3.5%); with work permit includes Temporary Permit of Permanence (85.3%), resident (2.1%), family of resident (0.7%), refugee (2.8%), and immigrant (3.5%)

^g^Political-administrative regions are according to the Government of Venezuela (June 7, 1969)

^h^Any combination of more than one travel method

### Multivariate analysis

The crude and adjusted analyses carried are presented in Table 7. Both crude and adjusted analyses were carried out. Months in Peru, age, and variables that showed significant association in bivariate analysis were considered in the crude analysis. A significant association with respect to age was found, namely, a 2% increased chance of having a clinically significant SR score. No association was found in the unadjusted model with respect to IR.

**Table 7:** Crude and adjusted Poisson regression analysis of the dichotomized Interpersonal Relations and Social Role domains of surveyed Venezuelan immigrants in Lima, Peru (*N* = 143)

Interpersonal Relations									
	Crude Analysis			Adjusted Statistic Model^a^			Adjusted Epidemiological Model^b^		
**Variables**	**PR**	**CI (95%)**	*p*-value*	**PR**	**CI (95%)**	Valor p*	**PR**	**CI (95%)**	*p*-value*
**Age (each additional year)**	0.93	0.86–1.00	0.060	0.94	0.87–1.02	0.133	0.94	0.87–1.02	0.164
**Civil status**									
Without partner	1.00	Ref	Ref	1.00	Ref	Ref	1.00	Ref	Ref
With partner	0.17	0.02–1.20	0.075	0.22	0.29–1.67	0.143	0.22	0.03–1.80	0.158
**Months in Peru**	0.97	0.88–1.07	0.592	0.97	0.88–1.07	0.609	0.97	0.88–1.07	0.581
Social Role									
	**Crude Analysis**			**Adjusted Statistic Model**^a^			**Adjusted Epidemiological Model**^b^		
**Variables**	**PR**	**CI (95%)**	*p*-value*	**PR**	**CI (95%)**	*p*-value*	**PR**	**IC (95%)**	Valor p*
**Age (each additional year)**	1.02	1.00–1.05	**0.033**	1.03	1.00–1.05	**0.033**	1.03	1.00–1.07	0.076
**Months in Peru**	1.01	0.95–1.08	0.710	1.01	0.95–1.08	0.683	1.01	0.94–1.08	0.821

*p < 0.050

^a^Adjusted by the principal independent variable (months in Peru), age, and any other statistically significant variable found in the bivariate analysis

^b^Adjusted for the confounding variables (migratory quality, monthly income, and civil status)

Two adjusted models were utilized in the adjusted analysis. The statistical model was adjusted for months in Peru, age, and variables that showed significant association in the bivariate analysis. The epidemiological model was adjusted for the confounding variables of migratory quality, monthly income, and civil status.

A significant association with respect to age was found in the adjusted statistical model, wherein a 3% increase in the chance of having a clinically significant SR score was observed. Correspondingly, the confounding variables of migratory status, monthly income, and civil status were included in epidemiologic analysis. However, no association was found in this model.

As with the crude multivariate IR analysis, no association was found in either adjusted model. As clinically significant SD and total scores were present in only a very small percentage of participants, no multivariate analysis was carried out with respect to these scores.

### Additional analysis

In order to estimate reliability, Cronbach’s alpha of the total OQ-45.2 score was calculated to be 0.87. Similarly, Cronbach’s alpha for the domains of IR, SR, and SD were 0.61, 0.64, and 0.85, respectively. SD showed good reliability while the other domains showed moderate reliability.

Two items were observed to have negative correlation with respect to the total score, while five had poor internal correlation. These questions were then excluded, and an exploratory factor analysis (EFA) was carried out, where eigenvalues greater than one and correlation values greater than 0.3 were considered. Six underlying factors were detected in the questionnaire. The first factor was the SD domain, while the other five factors did not correspond to the other two domains.

### Qualitative results

#### Characteristics of informants

A total of 16 informants were interviewed, each receiving a pseudonym, for purposes of confidentiality, as seen in Table 8. Informants were evenly divided between men and women and ages ranged from 19 to 49 years old. Time of residence in Peru ranged from two to 18 months. Thirteen informants had a partner, 14 had at least some higher education, and 12 informants held a valid work permit. The most common point of entry was Tumbes with 10 informants followed by Lima/Callao with five, and one through Piura. Bus was the most frequently used method of travel; just one informant each entered by airplane and on foot.

**Table 8:** Profile of in-depth interview informants (*N* = 16)

Pseudonym	Age	Months in Peru	Home state	Occupation	Education level
**“Roberto”**	25	16	Táchira	Sales representative	Higher education
**“Samuel”**	30	16	Capital District	Call center operator	Higher education
**“Pablo”**	19	11	Nueva Esparta	Delivery driver	Incomplete higher education
**“Angela”**	26	2	Carabobo	Homemaker	Higher education
**“Mariana”**	29	2	Anzoátegui	Food stand vendor	Incomplete higher education
**“Ingrid”**	26	4	Portuguesa	Homemaker	High school
**“Micaela”**	28	12	Trujillo	Post-partum period, not searching for employment	Higher education
**“Juan”**	38	9	Capital District	Car washer	Higher education
**“Franco”**	35	8	Monagas	Car washer	Higher education
**“Adriana”**	23	6	Anzoátegui	Clothes vendor	Higher education
**“Carlos”**	38	18	Bolívar	Watchman	Higher education
**“Ana”**	24	14	Carabobo	Sales representative	Higher education
**“Yesenia”**	31	18	Capital District	Information technician	Higher education
**“Natalia”**	21	13	Zulia	Clothes vendor	Higher education
**“Chris”**	23	8	Lara	Beggar	Incomplete higher education
**“Antonio”**	49	14	Carabobo	Watchman	Higher education

A majority of informants worked in the sales sector and were in the process of obtaining their TPP. A quarter of the sample was obtained from the mothers accompanying their children in the pediatrics department of a public hospital.

#### Self-perception of symptom distress

The symptoms of distress mentioned in most interviews were described in unspecific terms like “anxiety”, “psychological blow”, and “tension”. As one informant (Samuel, 30; pseudonym) phrased it, the information relayed from Venezuela, or lack thereof, affected him psychologically:

“Communication in Venezuela, due to internet problems, is also difficult. I have gone as long as two weeks without hearing from them. And when I do talk to them it is always the same difficulties. The psychological blows are strong. The deterioration in Venezuela has been happening exponentially, you see. Things get worse every time.”

Another contributing factor was the current situation in the country, especially the economic situation. While discussing this topic, an informant (Angela, 26; pseudonym) described how she was, “anxious to be better, to progress, to grow, to have better living conditions for my children.”

As for the manifestations of distress, a couple of informants described how their anxiety caused weight gain. In these cases, the individual had previously lost weight due to poor nutrition in Venezuela. With the newfound availability of food in Peru, one informant talked about how it was a way they managed their anxiety.

One informant mentioned how insomnia and headaches were a problem since arriving in Peru and attributed it to their state of anxiety and tension. Another informant with a previous history of depression mentioned how insomnia had presented before their migration and had continued during their time in Peru. Another informant (Ana, 24; pseudonym) detailed how memories and thoughts of life in Venezuela had become a recurring theme in her dreams and did not allow her to sleep well:

“I always dreamt the same. I started getting better a while ago. I started controlling it to get better, but I was dreaming practically the same thing every day. My recurring dream was that I would get to Venezuela, but did not have money to return to Peru. So, I would be in anguish because I would remember the chaos from back home and that wouldn not allow me to rest.”

A minority of participants mentioned frequent weeping and associated it with a constant state of anxiety and tension due to employment issues and news from Venezuela. In particular, one informant (Micaela, 28; pseudonym) had decided to leave Peru at the time of the interview. In tears, she discussed how she became pregnant after entering a relationship with a Peruvian a few months after arriving. During her pregnancy, the relationship ended and communication with the father of her child ceased. Feeling alone in a new country, she suffered from weeping and emotional lability stemming from job insecurity. Specifically, she (Micaela, 28; pseudonym) was afraid of losing her job due to a combination of being pregnant and vulnerability as a Venezuelan immigrant: “More than anything else, I was crying. It was more, I did not want to leave home, I wanted to stay and rest. Or when I was pregnant and on my tenth hour of work, I wanted to sleep. I felt bad and I could not say anything.”

One informant (Roberto, 25; pseudonym) described how his new fast-paced life in Lima had physical repercussions which he described as “tics on my face” from having to rush through the day.

However, not all topics mentioned with respect to distress were negative. A minority of informants brought up the fact that they had more energy in Peru. These informants narrated how their change in lifestyle had forced them to change the way they faced adversity. In particular, one informant (Yesenia, 31; pseudonym) with a history of major depressive disorder had decided to leave Venezuela in an attempt to overcome her condition. She went on to describe how she continued taking her medication at first, but, with time, discovered she no longer needed it and discontinued pharmacologic treatment.

### Self-perception of interpersonal relations

All informants mentioned having witnessed or received insults, whether on social networks or in public on account of being Venezuelan. One informant reported having been physically assaulted by a Peruvian on a public bus. However, some informants believe that the number of Peruvians with “xenophobic conduct” was a minority, similar to how they perceived the Venezuelans who commit crimes to be few. The majority attributed the xenophobic behavior to the “generalization” of delinquency to all Venezuelans as a whole.

One informant (Juan, 38; pseudonym) interpreted how the crimes committed by his compatriots affected him, causing him embarrassment as he stated:

“We are not here because we want to be, we are here out of necessity. I am going to apologize for my Venezuelan compatriots who have come here to do bad things, but we are not all the same. I apologize if you have been offended by some Venezuelans, because we have the mentality of work ethic and giving our children an education [sobbing].”

An informant (Yesenia, 31; pseudonym) brought up advice from her coworkers in Venezuela against emigrating to Peru due to a “retrograde mentality” instilled in Peruvians. After her time spent in Peru, she agreed with the assessment. As she stated, this mentality generated discomfort and influenced her decision against establishing herself in Peru in the long term:

“Really, almost all of my coworkers migrated to Europe, Argentina, Chile, Mexico, the United States. When I told them I wanted to come here, they said, ‘No, God, their mentality is a little bit backwards.’ How can I put it? … ‘They do not care about learning about other cultures, they are a bit closed off in their own thing.’”

A majority of informants touched upon the differences between Venezuelans and Peruvians with respect to manners, stating that Peruvians were less courteous and gracious. As one informant (Ana, 24; pseudonym) described:

“You arrive at some place and say, ‘good day’ and their response is, ‘OK’. ‘OK’ is not an answer to a greeting. Here you say, ‘good evening’ and nobody responds. You go on a bus, give up your seat, and nobody thanks you. That basic education that you learn at home, you do not see here. I would not want to have a child here and have him grow up with those manners.”

Likewise, some informants also detailed characteristics they believed to be Peruvian, such as “bitter”, “hermetic”, “dry”, and “stressed out”. In comparison, informants emphasized Venezuelan character as “open”, “warm”, “kind”, and “good-humored”. According to some informants, this perceived difference meant a more challenging adaptation to Peru. Given that this complicates relations between Peruvians and Venezuelans, the opinion was that this promoted isolation of social circles based on nationality.

However, nearly half of informants counted Peruvians amongst their friends; the other half-maintained friendships principally with Venezuelans. One informant considered Peruvians to be “receptive” and “warm”, this representing a main reason for selecting Peru as a destination country. In addition, nearly all informants felt that they had received help from Peruvians, mainly in the form of home gifts and food, but also emotional support. In a few cases a Peruvian had served in a “paternal” role and offered “familial” treatment.

More than half of informants reported having received support from fellow Venezuelans, generally, family members, friends, or partners. Here, the support included lodging and employment opportunities. Crucially, a majority had family waiting for them in Peru when they first arrived. This presence of family was a defining factor in selecting Peru as their destination country. Despite the feeling of support, one informant (Pablo, 19; pseudonym) believed that jealousy existed within Venezuelan compatriots of different socioeconomic levels. The same informant preferred Peruvian friendships because he was able to avoid conversations over struggles common in Venezuelans. One of these struggles was money, as he stated: “I do not talk with any Venezuelan. Imagine you are depressed because of your problems. You do not have money to send to your mother. Another Venezuelan comes along and says, ‘Oh, neither do I.’”

Every informant that did not live with members of his/her nuclear family mentioned that their absence was a source of “sadness” or “sorrow”. Nearly every informant talked about how their principal motivation for their emigration to Peru was the economic support they could provide their families. However, one informant (Yesenia, 31; pseudonym) did register a desire to “live outside of Venezuela” as their main reason.

A few informants manifested the discomfort caused by the suspicion Venezuelans paid more for rent, food, and clothes. Despite the many difficulties faced in Peru, several informants highlighted the fact that they had attained greater economic stability which allowed them to take better care of their family. This was a source of happiness which would have been difficult to obtain in Venezuela.

### Self-perception of social role

Among the most discussed topics pertaining to social role was labor exploitation, low wages, and absence of free time. A majority of informants declared their priority upon arrival in Peru to be employment. Notably, no informant regretted their decision to leave Venezuela and no informant regretted selecting Peru as their destination country despite the difficulties encountered at their workplace.

Every informant considered Peru as an intermediate country and had plans to leave the country. The majority planned to return directly to Venezuela, however, it had to be a Venezuela similar to the country before its socio-political and economic downturns. As one informant (Carlos, 38; pseudonym) described: “I would like it to be the Venezuela I miss, with security and respect for the law, without corruption, with the elimination of delinquency. A country where everything is accessible: education, health, and food.”

A minority did plan on moving on to another country, adding that they had family or friends in countries like Chile and Spain. This presence motivated them to consider these countries. Just like the majority of informants, this group had the goal of ultimately returning to Venezuela when it recovered. A single informant (Pablo, 19; pseudonym) manifested his rejection to returning to Venezuela. As he stated: “I always had it ingrained that when I was 30 years old, I would not be in Venezuela any longer. I simply want to use Peru as a trampoline to study then move to another country. Maybe Argentina, I am also Argentinean.”

With respect to employment, a majority of informants summarized the differences between both countries as “culture shock”. These informants believe that there are shorter work hours, less workload, and a greater amount of free time in Venezuela. They interpret that employers base a workday on tasks, which require overtime, rather than hours. Furthermore, there is no additional pay for overtime or holidays. As one informant (Yesenia, 31; pseudonym) discussed:

“They go to the bathroom with labor laws. They require you to work extra hours on tasks that they know you cannot finish in a normal day. If you do not finish, you do not get paid. Even small companies force you to work a minimum of 12 hours. The law is null here, you can forget getting paid double on holidays.”

More than half of informants reported uncomfortable experiences at work. A majority characterized the relationship they had with their bosses as hostile or authoritarian. Of the five female informants who held jobs at the time of the interviews, four had a negative relationship with their employer; a departure from male informants, where a small minority opined the same. However, some informants did enjoy a positive relationship with their employer. In particular, a young informant described in detail how his boss served as a paternal figure in Peru and even offered him free lodging in his house. In other instances, employers served as a support system, aiding in the adaptation process.

Some informants believed that the work conditions they suffered were due to their immigrant status while others considered it to be a part of the Peruvian work environment. It is worth highlighting that every informant who reported suffering from poor treatment denied that this was commonplace in Venezuela. As a result of 12-hour workdays, they did not have time to engage in activities outside of work. As one informant (Chris, 23; pseudonym) recounted: “They liked to order us around at my old job, the same place that fired us. They wanted everything to be done quickly, perfectly, without a single mistake because everything was an earful otherwise. Something we are not accustomed to.”

One particular case involved a 23-year-old informant (Adriana, 23; pseudonym) who relived an occasion of sexual harassment by a previous employer. She described him as “touchy” which led to her resignation. The same informant described an instance of groping in public by a Peruvian man, a situation she said she never suffered in Venezuela and one which deeply affected her. In tears, she stated: “A Peruvian man approached and followed me … he groped my rear … it was horrible. It was horrible, never in my life had that happened. I wanted to cry because I was not able to do anything.”

At the time of interview, not all informants were employed, but no informant was searching for employment. However, a majority did have experience with unemployment in Peru, whether personal or of a partner. In the opinion of the informants, the average time they had spent looking for employment varied from one to 2 weeks. A majority of informants also manifested that their monthly income was below the minimum wage. Similarly, around a third of informants could not work in their profession as a result of the inability to transfer their set of skills in Peru.

A couple of housewife informants lamented the absence of the child-rearing support system they could count on in Venezuela. As one informant (Micaela, 28; pseudonym) mentioned: “Here, I do not have my own home. I have to pay rent and depend on a host of other factors. At least in Venezuela, I had a place of my own. And my family is over there, I am alone here.” In contrast to those informants without children, those with children had to prioritize financial resources for their children rather than family back in Venezuela.

Additionally, more than half believed that employers viewed them as a source of “cheap labor”. Two informants reported receiving counterfeit money as payment. As one informant (Juan, 38; pseudonym) put it: “I had to work 17 hours in my first job here in Peru and I was paid with a counterfeit 200 soles bill. That was my first disappointment here.”

## Discussion

### Main findings

This mixed-methods study, which evaluated the distress in Venezuelan immigrants in Peru, found that 4.2% of the surveyed population scored above the cutoff point. No association was found between SD and time in Peru. Gender, however, was associated with a clinically significant overall score. With respect to other domains, associations between IR and age and IR and civil status were found. Another association was SD with age group. In the adjusted model, a 3% increase in having a clinically significant SR score was found for each additional year of age.

### Comparison with other studies

The present study did not find an association between time in Peru and self-perception of distress. In contrast with published literature, our study measured time in months while other studies measured time in years. One such study explored established immigrant populations and concluded that the, mostly Peruvian, immigrant population in Chile had lower rates of mental health disorders when compared to native Chileans [4]. This aligns with the concept of “immigrant paradox” [8].

Our study found an association between gender and overall OQ-45.2 score. Indeed, the association between gender and mental health has been reported previously in a systematic review of mental health in immigrants in Spain. Herein it was reported that women showed lower levels of mental health status than men [33]. Other studies carried out in Switzerland and the United States also reported this difference [8, 34]. The association observed in this study concurs with published literature around the world.

The association between age group and SD has been reported in other studies. However, one difference between this study and other literature is that we reported only in adults. One review described how psychiatric disorders were less prevalent in immigrants who arrived in the United States after 14 years of age when compared to those who arrived prior to that age [8]. A Swedish study found that mortality in immigrants was lower in those who arrived before 18 years of age [35]. This suggests that arriving in a country at a younger age equates to rates of mental health disorders similar to the receiving population.

Civil status was associated with difficulties stemming from IR in Venezuelan immigrants. This result is similar to a Swedish study where being divorced or widowed was a risk factor for presenting anxiety, depression, and low levels of self-perceived wellbeing [36]. Another Swedish study found that civil status was associated with mental health problems as measured by psychotropic drug consumption [37]. However, no studies that directly associated civil status with interpersonal relationship problems in immigrant populations were found.

With respect to the multivariate analysis, it was found that each additional year of age corresponded to a 3% increase in the probability of having symptoms arising from SR. No article that associated additional years of age with SR was found in our literature search. However, there are studies that have evidenced mental health deterioration until reaching a level akin to the native population [5]. Among the factors which influenced mental health deterioration were employment status and income, which are encompassed in the SR domain [38].

Cronbach’s alpha in the OQ-45.2 was found to be good. However, it was lower than Cronbach’s alpha reported in the validation to the Spanish language [24]. Cronbach’s alpha in SR and IR was moderate, while in SD it was acceptable. It was also found that OQ-45.2 had good reliability in SD.

With respect to IR, our study concurs with a study carried out by Oxfam which found that Venezuelan immigrants in Peru frequently experience or witness xenophobic conduct [39]. Likewise, placing the blame on the Venezuelan population for crime coincides with a qualitative Peruvian study [40]. The aforementioned study concluded that the blame was conducive to the development of discrimination against Venezuelans in Peru.

The present study showed how the separation of the family nucleus was a prevalent stress factor on a migrant, a finding similar to a Canadian study. The authors concluded that family separation strongly influenced emotional distress on migrants, and also led to the prioritization of family reunification [41]. An American study inferred that post-migration family separation represented one of the main causes of distress in immigrants [42]. The most prevalent post-migration stress factors in refugees were inadequate housing, financial difficulties, and family separation [43].

The fact that every informant had a male boss has been previously described in studies, and places women in a state of vulnerability [44, 45]. Furthermore, the incident of groping and sexual harassment is similar to the findings in a study by Oxfam [39]. Herein the conclusion was that Venezuelan women faced higher risk of abuse, sexual exploitation, and gender-based violence. Another study came to the conclusion that migrant women were particularly vulnerable to sexual harassment; the main factor was having a male coworker [46] 6.

In the SR domain, one of the most discussed topics was the poor working conditions compared to Venezuela. Nearly all female informants had a poor relationship with their boss, a finding that has been previously described [47]. The authors reported that women had higher chances of suffering sexual violence and harassment because they have greater dependence on their employers than do men. The same study also found that the loss of social roles could serve as a source of mental health alterations [47]. This finding is convergent with our study where we observed that the loss of social role is, at least partly, a result of changes experienced at work. Another point of convergence is the perceived lack of support in child-rearing by mothers.

A qualitative study carried out in Italy and Spain found that the majority of Indian immigrants were economic migrants [48]. Our qualitative component consisted of 16 informants, all of whom migrated to Peru in search of economic growth. Similar to this study, we found that Venezuelan immigrants are influenced and motivated by the experiences and suggestions of their compatriots who reside in other countries [48]. On the other hand, a Portuguese study observed that more than half of immigrants planned on staying in the long term. This was considered by the authors to be a decisive factor for integration into Portuguese culture [49, 49]. Contrastingly, no informant in our study imagined their long-term future in Peru.

A qualitative Spanish study exposed the inability to work in their professional field as well as their condition as an immigrant as barriers for immigrants to access adequate work conditions; a finding reflected in our own study [50]. These factors may contribute to the formation of mental health problems.

While no qualitative studies exploring SD in immigrants have been found, there are quantitative studies on the subject. A Swiss study described how immigrants had alterations of sleep which included difficulty falling asleep and had higher prevalence of interrupted sleep than the non-immigrant population [51]. When considering the Peruvian population, a cross-sectional study found that around 30% of the supposedly healthy population suffered from somnolence with the percentage for insomnia hovering above 40% [52]. Less than a quarter of informants reported insomnia, but nearly all reported feeling tired or using free time to sleep.

One prevalent topic during interviews was weight gain. A qualitative study in the United States with 25 Latina immigrants found that weight gain was a result of changes in lifestyle stemming from the migration process, namely, depression and stress [53]. After 20–30 years in Canada, the percentage of immigrants who were overweight or obese was equal to or greater than the corresponding percentage of native Canadians [54]. Of note, the weight gain seen in the Venezuelan population could be recuperation of lost weight due to the food shortages in Venezuela mentioned by informants. Other studies of Hispanic immigrants to the United States describe eating disorders and their association with the migration process as well as the connection between Hispanic culture and food [55–57].

While many studies focused on diagnosing mental disorders in immigrants, this study did not use an instrument with this goal in mind. A systematic review in Sweden detailed how immigrants have a higher risk for mental disorders, with particular attention to depression and anxiety, than the native population [58].

A mixed-methods study carried out in the immigrant Chinese population in Birmingham, UK, described the difficulties the population faced when filing legal documents as a source of mental health problems [59]. This, however, was not a source of problems in the population of interest in this study. Most had or were in the process of acquiring their work permit and did not describe major difficulties in doing so. Another process mentioned by informants was signing up for the Peruvian health system; however, it was not described as a source of difficulties unlike other studies [60–62].

### Global health relevance

Only Colombia has received more Venezuelan immigrants than Peru, studies there have shown that the immigrant Venezuelan population is at higher risk for major depressive disorder and generalized anxiety disorder [63]. As such, governing bodies and non-governmental organizations that oversee immigrant rights and interests have described the need for community outreach through psychosocial support systems and screening in search of stressors as steps necessary to prevent psychiatric disorders.

In 2017, the Global Compact for Migration established 23 objectives to achieve ordered, regular, and safe migration. The ones relevant to the scope of this study and which need to be addressed by Peruvian authorities deal with ensuring legal identity of migrants, safeguarding decent work conditions, empowering migrants and societies with the aim of inclusion and cohesion, and investing in skills development and recognition of skills and qualifications [64].

### Strengths and limitations

The mixed-methods nature of this study is a strength which allows us to combine quantitative and qualitative data to better approach the dynamic migratory process. The in-depth interviews, in particular, give a voice to the informants beyond close-ended questions and permits them the opportunity to make an interpretation of their experiences in Peru.

However, the main quantitative component has certain limitations like the non-probabilistic sampling method. In order to help offset this limitation, we collected data in different areas of Lima in an attempt to homogenize the sample. Finally, as a cross-sectional study, data collection took place at a single point, and, as a result, causality cannot be detected or attributed.

Another strength of this study was the calculation of internal consistency and the exploratory factor analysis that we carried out. We found that the OQ-45.2 had a good Cronbach’s alpha, measures the SD domain satisfactorily, but needs to have some questions in the other domains adjusted for the immigrant Venezuelan population in Peru.

As for the qualitative component, data cannot be extrapolated to the entire immigrant Venezuelan population as it only describes the migratory experience and interpretation of the informants. Additionally, convenient and snow-sampling under over represents immigrants from better social conditions and underrepresents the whole immigrants Venezuelan living in Peru. Of note, the informants were interviewed in a variety of situations which could each reflect differently on their levels of stress. Moreover, the number of qualitative studies focusing on immigrants is far fewer than the number of quantitative ones which stresses the importance of giving them a voice outside of close-ended questions.

## Conclusions

A minority of participants registered a clinically significant total and domain score. No association between months in Lima and the self-perception of distress was found. However, this could be due to the short amount of time spent in Peru and any change in self-perception might only be perceived after years or decades spent in Peru. An association that was observed was the increase in the risk of clinically significant SR score with additional years of age. A variety of distress symptoms was mentioned by informants, which included symptoms of anxiety and alterations.

### Recommendations

Given the short time since the beginning of the present migration phenomenon, we suggest carrying out a greater number of mixed-methods or qualitative studies centered on distress, its manifestations, and resulting mental disorders in the Venezuelan population. Also, given the recent context of COVID-19, a study on the impact of the repercussions of the pandemic on vulnerable populations would be pertinent.

## Data Availability

The datasets generated and/or analyzed during the current study are not publicly available due to the sensitive information they contain. Transcriptions of the in-depth interviews also contain sensitive information, such as personal information of the informants. However, both are available on reasonable request from the corresponding author.
